# The effect of bone marrow mesenchymal stromal cell exosomes on acute myeloid leukemia’s biological functions: a focus on the potential role of LncRNAs

**DOI:** 10.1007/s10238-024-01364-6

**Published:** 2024-05-22

**Authors:** Sahar Jalilivand, Mehrdad Izadirad, Nader Vazifeh Shiran, Ahmad Gharehbaghian, Sina Naserian

**Affiliations:** 1 Department of Laboratory Hematology and Blood Bank, School of Allied Medical Sciences, Shahid Beheshti University of Medical Sciences, Tehran, Iran; 2 CellMedEx, Saint Maur des Fossés, France

**Keywords:** Acute myeloid leukemia, Exosome, Bone marrow mesenchymal stromal cells, LncRNA

## Abstract

Acute myeloid leukemia represents a group of malignant blood disorders that originate from clonal over-proliferation and the differentiation failure of hematopoietic precursors, resulting in the accumulation of blasts in the bone marrow. Mesenchymal stromal cells (MSCs) have been shown to exert diverse effects on tumor cells through direct and indirect interaction. Exosomes, as one of the means of indirect intercellular communication, are released from different types of cells, including MSCs, and their various contents, such as lncRNAs, enable them to exert significant impacts on target cells. Our study aims to investigate the effects of BM-MSC exosomes on the cellular and molecular characterization of HL-60 AML cells, particularly detecting the alterations in the expression of lncRNAs involved in AML leukemogenesis, cell growth, drug resistance, and poor prognosis. BM-MSCs were cultured with serum-free culture media to isolate exosomes from their supernatants. The validation of exosomes was performed in three stages: morphological analysis using TEM, size evaluation using DLS, and CD marker identification using flow cytometry. Subsequently, the HL-60 AML cells were treated with isolated BM-MSC exosomes to determine the impact of their contents on leukemic cells. Cell metabolic activity was evaluated by the MTT assay, while cell cycle progression, apoptosis, ROS levels, and proliferation were assessed by flow cytometry. Furthermore, RT-qPCR was conducted to determine the expression levels of lncRNAs and apoptosis-, ROS-, and cell cycle-related genes. MTT assay and flow cytometry analysis revealed that BM-MSC exosomes considerably suppressed cell metabolic activity, proliferation, and cell cycle progression. Also, these exosomes could effectively increase apoptosis and ROS levels in HL-60 cells. The expression levels of p53, p21, BAX, and FOXO4 were increased, while the BCL2 and c-Myc levels decreased. MALAT1, HOTAIR, and H19 expression levels were also significantly decreased in treated HL-60 cells compared to their untreated counterparts. BM-MSC exosomes suppress cell cycle progression, proliferation, and metabolic activity while simultaneously elevating the ROS index and apoptosis ratio in HL-60 cells, likely by reducing the expression levels of MALAT1, HOTAIR, and H19. These findings suggest that BM-MSC exosomes might serve as potential supportive therapies for leukemia.

## Introduction

Acute myeloid leukemia (AML) is one of the most prevalent types of malignant hematological diseases. In this condition, the excessive and abnormal proliferation and the differentiation inability of stem cells and other blood precursors lead to an increase in the accumulation of blasts in the bone marrow (BM) [1, 2] and their entry into the bloodstream. This disease is often seen in adults with an average age of over 60 years [3, 4]. The efficacy of treatment and its outcome are contingent upon various factors, such as age, mutations, and recurrence. Despite recent therapeutic advances, the management of AML remains challenging, with a notably high mortality rate [5, 6].

Among the numerous types of stem cells, mesenchymal stromal cells (MSCs) hold a prominent position as one of the most indispensable sources of cell therapy and other clinical applications due to their unique and practical characteristics, such as the multi-lineage capacity to differentiate into various types of cells [7]. These cells can modulate numerous tumor mechanisms through either cell-to-cell interactions or the secretion of a wide range of factors such as growth factors, cytokines, extracellular vesicles, and more, leading to the progression or abrogation of tumorigenesis [8]. In the case of leukemia, researchers have demonstrated the inhibitory effect of MSCs on leukemic cell proliferation and differentiation [9].

Exosomes are a set of extracellular vesicles (EVs) ranging in size from 50 to 100 nm that were first discovered in 1983 and play a crucial role in intercellular communication [10, 11]. They are secreted from different types of cells and are widely present in both extracellular environments and body fluids [10, 12]. Exosomes harbor diverse cargoes of nucleic acids, proteins, lipids, and other biologically important molecules. The composition of these cargoes varies depending on the cell and tissue which the exosomes originate from and can exert efficacious effects after being absorbed into the target cell [10]. Numerous studies have indicated that MSC-derived exosomes have a similar function as their parent cells in affecting tumor survival, growth, metastasis, and drug response [13–16].

Long non-coding RNAs (LncRNAs) account for transcripts with a length of more than 200 nucleotides with either negligible or restricted protein-coding potential [17]. Despite their generally lower expression levels, compared to mRNAs, lncRNAs are proven to be a critical part of the functional transcriptome, and their low levels of expression do not impede their functions [18]. They are believed to play various roles in many biological processes, including gene expression, cell signaling, and DNA repair [19]. Additionally, lncRNAs have a role in inducing nucleosome modification and chromatin remodeling, regulating variable splicing modes and protein activity, creating endogenous siRNAs, and modifying protein localization [20]. A number of articles have mentioned their abnormal expression in many cancers, including hematological malignancies, which affect different stages of disease progression, such as proliferation, apoptosis, metastasis, and even drug resistance [17, 21–23].

H19, Metastasis-associated lung adenocarcinoma transcript 1 (MALAT1), HOX Transcript Antisense RNA (HOTAIR), and Taurine-Upregulated Gene 1 (TUG1) are considered as poor-prognosis lncRNAs whose aberrant overexpression has proliferative and anti-apoptotic effects on AML cells and is correlated with higher BM blast counts, poor chemotherapy response, and shorter overall survival (OS) in AML patients [20, 24, 25].

Our study aimed to investigate the effect of BM-MSC exosomes on some of the biological functions of HL-60 AML cells, such as viability, proliferation, apoptosis, and Reactive Oxygen Species (ROS) levels, which have not been widely reported as relevant biological parameters, and to explore whether these exosomes could regulate the expression of MALAT1, HOTAIR, H19, and TUG1 lncRNAs.

## Materials and methods

### Cell culture and identification

The human BM-MSCs of a healthy 26-year-old male donor were obtained from Royan Institute for Stem Cell Biology and Technology (Tehran, Iran) at passage 1, which is the first culture of BM-MSCs after being transferred from BM to a fresh growth medium in the flask. The BM-MSCs were cultivated in minimum essential medium Eagle, alpha modification (*α*-MEM) (Bioidea, Tehran, Iran) supplemented with 7% fetal bovine serum (FBS) (Gibco, Brazil), 1% L-glutamine, and 1% penicillin/streptomycin. The culture media were refreshed every 3–4 days, and at 80–90% confluence, the adherent cells were passaged using 0.25% trypsin–EDTA (Gibco, Canada).

The HL-60 AML cell line was selected as a model of AML cells because, according to previous studies [24–27], it has a higher expression of MALAT1, HOTAIR, H19, and TUG1 lncRNAs than other AML cell lines. In addition, the impact of BM-MSC exosomes on some biological functions of AML has not been previously investigated in this cell line. HL-60 cell line was purchased from the Pasteur Institute (Tehran, Iran) at passage 15 and cultured in RPMI-1640 medium (Gibco, USA) supplemented with 15% FBS, 1% L-glutamine, and 1% penicillin/streptomycin. All cells were incubated at 37 °C with 5% CO_2_.

To characterize BM-derived MSCs, their morphology (passage 2) was verified under the inverted microscope, and the expression of common MSC surface markers (CD45, CD90, CD14, CD105, CD34, and CD73) was analyzed by BD FACS Calibur (BD Biosciences, San Jose, CA, USA).

### Isolation of BM-MSC exosomes

Exosomes secreted by BM-MSCs were extracted from their conditioned medium using a precipitation method. BM-MSCs were seeded into a T75 culture flask at passage 3–6. When the cells reached to 90% confluency, the culture medium was removed, and the cells were washed with phosphate-buffered saline (PBS) 1X (Sigma, USA). After discarding the PBS 1X, 10 mL of serum-free *α*-MEM was added to the flask, and the cells were cultured for 72 h before the conditioned medium was harvested. It was then centrifuged at 3000 × g for 10 min to drop cells and cellular debris. In order to purify the exosomes, the exosome precipitation reagent (Exocib, Cibbiotech, Iran) was used. The mixtures were vortexed for 5 min and incubated at 4 °C overnight. Subsequently, the mixtures were centrifuged for 40 min at 3000 × g to precipitate exosomes. The final exosome pellets were re-suspended in PBS 1X and stored at −20 °C for downstream analysis.

The flow cytometry was used to confirm the phenotype of exosomes by detecting the expression of CD63, CD9, and CD81 markers.

### Dynamic light scattering (DLS) measurement

To evaluate the size of exosomes, the DLS method was performed by a Malvern Nano ZS instrument (UK). The samples were diluted with 100 μL of PBS 1X and aliquoted into a quartz cuvette, and absorption was measured at a wavelength of 630 nm. The results were analyzed with Zeta Sizer software (v. 7.11).

### Transmission electron microscopy (TEM)

The morphologic characteristics of BM-MSC exosomes were observed by TEM (A Zeiss-EM10C-Germany). To prepare the samples, 25 μL of diluted exosomes were carefully placed on carbon films on 300 mesh copper grids (AGS160-3) and incubated for 3 min at room temperature (RT). Subsequently, the exosomes were stained with a uranyl acetate solution (PELCO, Ted Pella). Given the dehydration and fixation of exosomes during the preparation process, the shape and size of exosomes could be changed in the captured image.

### Measurement of the exosomes' protein content

To quantify the concentration of exosomes, the protein content was measured by bicinchoninic acid (BCA) assay. Based on the protein assay kit (KIAZIST, Iran), negative and positive controls, standard and unknown sample dilutions were prepared, and then, the absorption was measured at 570 nm. The standard chart was drawn using a specific concentration of bovine serum albumin (BSA) protein, and the concentration of the unknown samples was measured according to this chart.

### Cell viability assay

To measure the cell viability of the HL-60 leukemic cell line after treatment with MSC exosomes, the MTT assay was performed. HL-60 cells were seeded (12 × 10^3^ cells/well) in a 96-well plate using RPMI culture medium (15% FBS, 1% penicillin/streptomycin) and incubated overnight at 37 °C in a 5% CO_2_ incubator. The cells were treated with prepared concentrations of MSC exosomes (0, 50, and 100 μg/mL, 3 wells × each dose), which is in accordance with doses used in similar articles [28], for 24 h and 48 h. Then, 10 µl of 5 mg/mL MTT reagent (Cibbiotech, Iran) was added to each well, and the plate was incubated for 4 h in a humidified 37 °C incubator. After incubation, the plate was centrifuged at 2700 × g for 20 min. To dissolve the formazan crystals formed in the wells, 100 µL of dimethyl sulfoxide (DMSO) (Cibbiotech, Iran) was added to each well and incubated for 15 min. The ELISA reader measured the optical density (OD) at a wavelength of 570 nm (Biotech ELX800, USA).

### Apoptosis assay

Apoptosis of leukemic cells was analyzed using an Annexin V-fluorescein isothiocyanate (FITC)/propidium iodide (PI) cell apoptosis detection kit (Sigma, Germany). Briefly, HL-60 cells were seeded in 12-well plates at a density of 12 × 10^4^ cells per well, and the plate was incubated overnight at 37 °C. After incubation, the cells were co-cultured with different concentrations of exosomes (0, 50, and 100 µg/mL, 3 samples × each dose), and after 24 h, the cells were harvested, centrifuged for 5 min at 1500 rpm, and washed twice with 2 ml of precooled PBS 1X to remove culture medium. After washing, 500 µl of 1X binding buffer was added to the cells. Then, the cells were mixed with 3 µL of Annexin V-FITC and incubated for 15 min at 4 °C in the dark. Subsequently, 1 ml of 1X binding buffer was added to the tubes and centrifuged for 5 min at 1500 rpm. Then, the binding buffer was discarded from the tubes, and another 500 µl of 1 × binding buffer was added to the cells again. Finally, 3 µL of PI was added to each sample, and the mixtures were analyzed immediately by BD FACS Calibur (BD Biosciences, San Jose, CA, USA).

### Cell cycle analysis

The cell cycle was examined using PI staining and analyzed by flow cytometry. HL-60 leukemic cells were seeded in 12-well plates at a density of 12 × 10^4^ cells per well, and the plate was incubated overnight at 37 °C. Then, the cells were treated with 0, 50, and 100 µg/mL of BM-MSC exosomes (3 samples × each dose) for 24 h. Subsequently, the cells were collected, centrifuged for 5 min at 1500 rpm, washed once with cold PBS 1X, and suspended in 50 μl of cold PBS 1X. To fix the cells, 1 mL of cold 70% ethanol was added dropwise to the cell suspension and vortexed gently. During fixation, the temperature of the ethanol was −20 °C. The cell suspension was centrifuged to discard the ethanol and washed with PBS 1X once again. PBS was gently removed, and 1 ml of PI Master Mix solution containing 40 μl PI, 10 μl RNase, and 950 μl PBS 1X was added to the cells. The cells were incubated for 30 min at RT before flow cytometric analysis.

### Reactive oxygen species assay (ROS assay)

HL-60 cells were seeded in 12-well plates at a density of 12 × 10^4^ cells per well, and the plate was incubated overnight at 37 °C before adding exosomes. After 24 h of treatment with 0, 50 and 100 μg/mL of BM-MSC exosomes (3 samples × each dose), the cells were collected and washed with 2 ml of PBS 1X, centrifuged for 5 min at 1500 rpm, and brought to a volume of 1 ml with fresh PBS 1X. 2 µl of DCFH-DA stain was added to the tubes, and the cells were incubated for 45 min at 37 °C in the dark. Subsequently, 1 ml of PBS was added to the samples and centrifuged for 5 min at 1500 rpm. The previously added PBS was discarded, and 500 µl of PBS 1X was added to the cells. Then, 5 µL of PI was added to each sample, and the ROS levels of the mixtures were assessed by BD FACS Calibur (BD Biosciences, San Jose, CA, USA).

### Ki-67 cell proliferation assay

The Ki-67 protein is a nuclear antigen related to cell proliferation that can be used as a marker for cell proliferation assays. To determine the effect of BM-MSC exosomes on HL-60 cells’ proliferation, first, HL-60 cells (seeded in 12-well plates at a density of 12 × 10^4^ cells/well), treated with 0 and 100 μg/mL of exosomes (3 samples × each dose) for 24 h at 37 °C, were collected, washed with 1 mL of PBS 1X and centrifuged for 5 min at 1500 rpm. After that, 100 μL of fixation reagent was added to the tube and incubated at RT for 30 min. The cells were washed twice with 1 mL of Perm/Wash solution (BioLegend, USA). Then, 100 μL of Perm/Wash solution along with Ki-67 antibody was added to the samples, incubated for 30 min at 4 °C, and washed twice with 1 ml of Perm/Wash solution again. The final step was analyzing samples with BD FACS Calibur (BD Biosciences, San Jose, CA, USA).

### RNA analysis, cDNA synthesis, and RT-qPCR assay

The total RNA of HL-60 cells treated with 0 and 100 µg/mL of exosomes (3 samples × each dose) for 24 h was isolated by 800 µl TRizol™ Reagent according to the manufacturer’s instructions (Qiagen, USA), and the RNA was reverse transcribed to cDNA according to the synthesis kit (Thermo Scientific, USA). To confirm the quantity and purity of extracted RNA, a NanoDrop was used (Thermo Scientific, USA) to examine the OD at 260 nm and the ratio of OD 260/280, respectively. As a housekeeping gene, GAPDH was used to normalize the expression levels of lncRNAs and mRNAs. Reverse transcription-quantitative polymerase chain reaction (RT-qPCR) was carried out using SYBR™ Green Real-time PCR Master Mixes (Amplicon, Denmark) and primers specific to the genes of interest (Table 1). The test was performed under the following conditions: denaturation at 95 °C for 5 min, followed by 40 cycles of 95 °C for 15 s, 60 °C for 15 s, and 72 °C for 15 s, and a final 10 min at 72 °C for extension. The Livak method (${2}^{-\Delta \Delta {\text{Ct}}}$) was applied to calculate gene expression fold changes.

**Table 1:** This table provides the primer sequences used in this study

Genes	primer	Sequences (5’– > 3’)
GAPDH	Forward Reverse	ATGGGGAAGGTGAAGGTCG TAAAAGCAGCCCTGGTGACC
MALAT1	Forward Reverse	GTAACGATGGTGTCGAGG CAGCATTACAGTTCTTGAAC
HOTAIR	Forward Reverse	GCTACTTGTGTAGACCCA ACCTTTGCTTCTATGTTCC
H19	Forward Reverse	AGCGTAGGGTCCAGCAC GAGCACTGCCTGTCTTCC
TUG1	Forward Reverse	AGGCTATCAGAATAACTACTCATCC ACTAGAATGGGTGTCTTCGTC
C-MYC	Forward Reverse	GCGACTCTGAGGAGGAAC TCTTGGCAGCAGGATAGTC
P21	Forward Reverse	CCAGCATGACAGATTTCTACC AGACACACAAACTGAGACTAAGG
BAX	Forward Reverse	CAAACTGGTGCTCAAGGC CACAAAGATGGTCACGGTC
BCL2	Forward Reverse	GATAACGGAGGCTGGGATG CAGGAGAAATCAAACAGAGGC
P53	Forward Reverse	AGCACTAAGCGAGCACTG CCTGGGCATCCTTGAGTTC
FOXO4	Forward Reverse	CACGTATGGATCCGGGGAAT CCCCTCCGTGTGTACCTTTTC

### Statistical analysis

All tests were performed in triplicate. Quantitative analysis of flow cytometry results was performed using FlowJo (BD, USA). Statistical data were analyzed using Graph Pad Software (Graph Pad Prism version 9.00), and total data were expressed as the mean ± standard deviation (SD). Paired t test was used to evaluate the significant difference between the two groups, while one-way and two-way ANOVA was used to assess the difference between multiple groups. The level of significance was considered as *p* < 0.05.

## Results

### Morphology and immuno-phenotyping of BM-MSCs

**Fig. 1:**
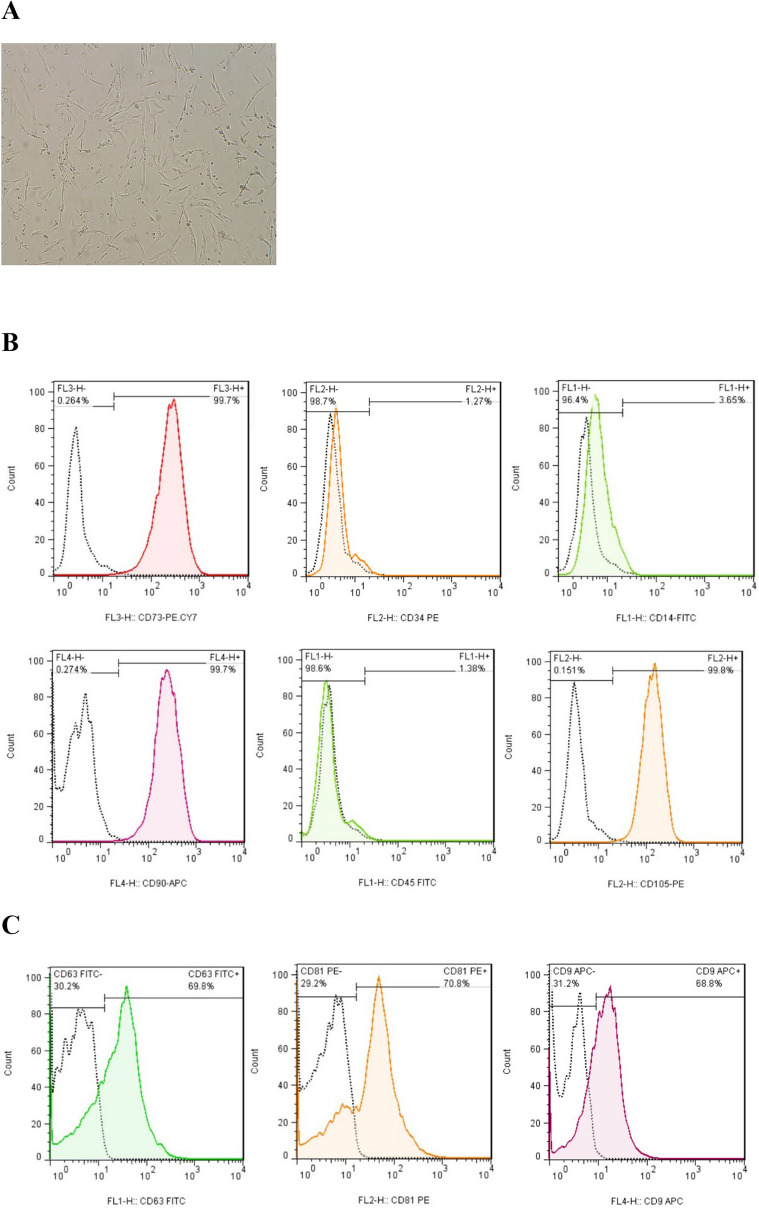

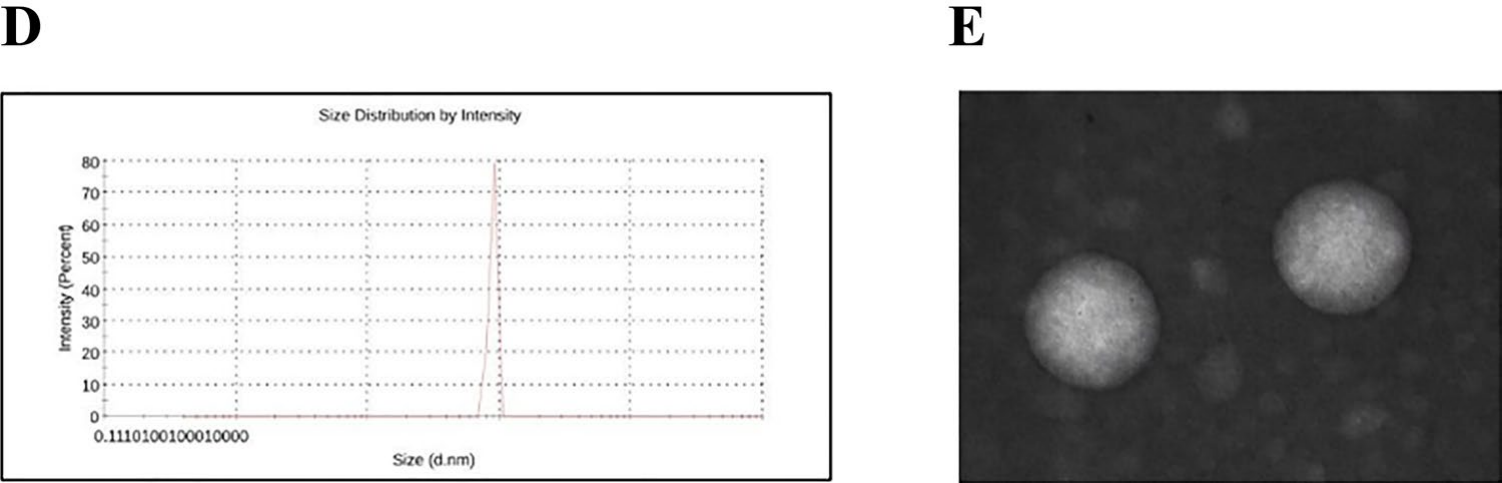
Confirmation and identification of BM-MSCs and isolated MSC Exosomes. (**A**) BM-MSCs at passage 2 exhibited the fibroblast-like morphology under an inverted microscope. (**B**) The immune-phenotype of BM-MSCs was checked by flow cytometry. BM-MSCs expressed CD73, CD90, and CD105, but lacked CD14, CD34, and CD45. (**C**) The expression levels of the exosomal surface markers CD9, CD81, and CD63 in BM-MSC exosomes were examined using flow cytometry. (**D**) The size of BM-MSC exosomes was measured using a Zetasizer. The exosomes’ size ranged from 80 to 100 nm. (E) Morphology of BM-MSC exosomes under TEM. Scale bar: 201 nm. The exosomes exhibited a spherical-shaped morphology

The BM-MSCs exhibited uniform spindle-shaped morphology in inverted microscopy, which implied that these cells adhered to the plastic flask (Fig. 1A). Flow cytometry immuno-phenotypic analysis revealed the expression of CD90, CD105, and CD73 on the surface of BM-MSCs and the lack of CD14, CD45, and CD34 expression, confirming that the cultured cells were MSCs (Fig. 1B).

### Characterization of isolated exosomes

Our results demonstrated that CD81, CD63, and CD9, exosome specific markers, were all expressed in exosomes isolated from BM-MSCs (Fig. 1C). Furthermore, the results of the DLS analysis shown in Fig. 1D indicated that the diameters of exosomes ranged between 80 and 100 nm. Accordingly, electron microscopy validated that the majority of these nanoparticles exhibited a round-shaped morphology, quite similar to exosomes (Fig. 1E).

Moreover, the exosomes’ concentration and protein content were assessed via BCA assay revealing a concentration of 1497.071 µg/mL.

### Effect of BM-MSC exosomes on the metabolic activity of HL-60 cells

**Fig. 2:**
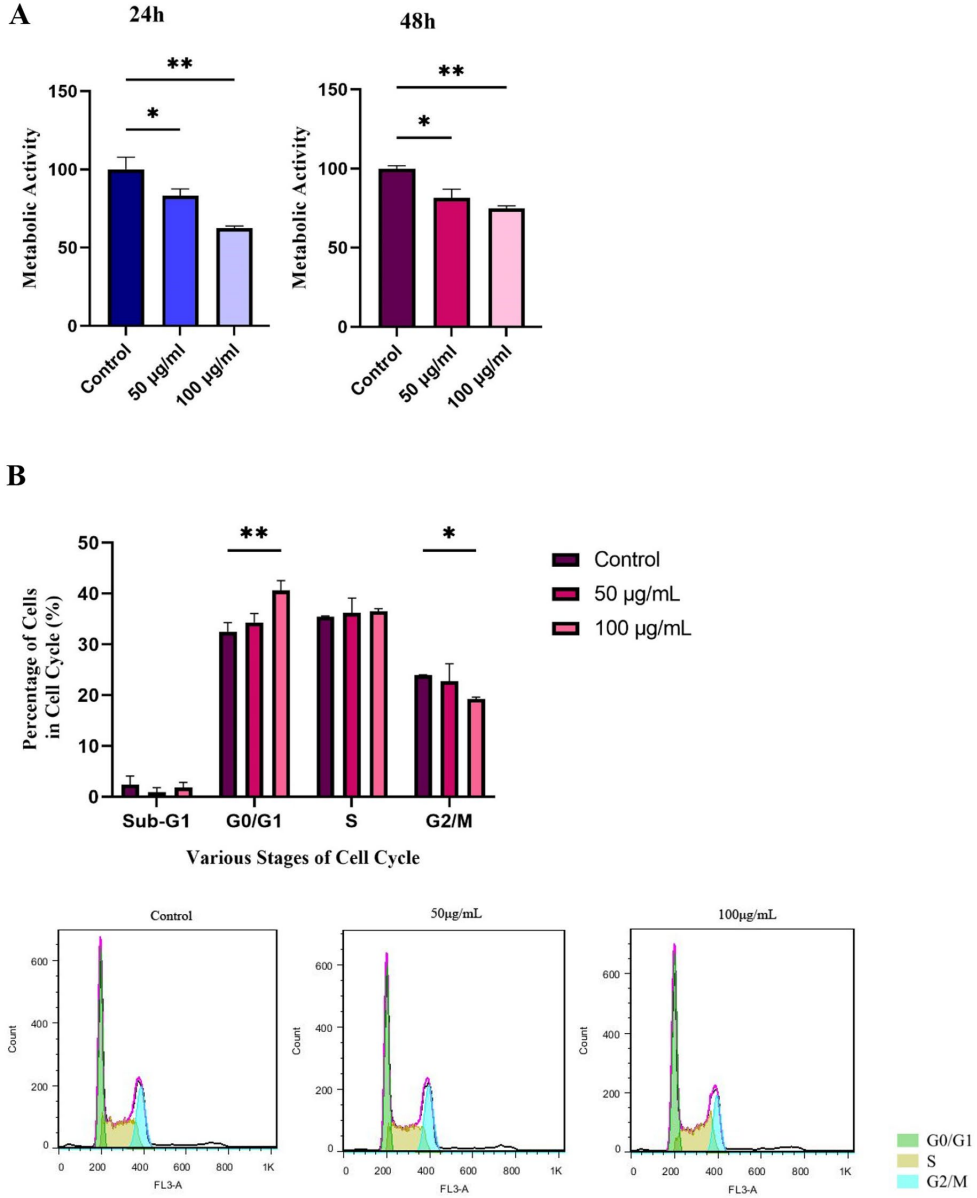

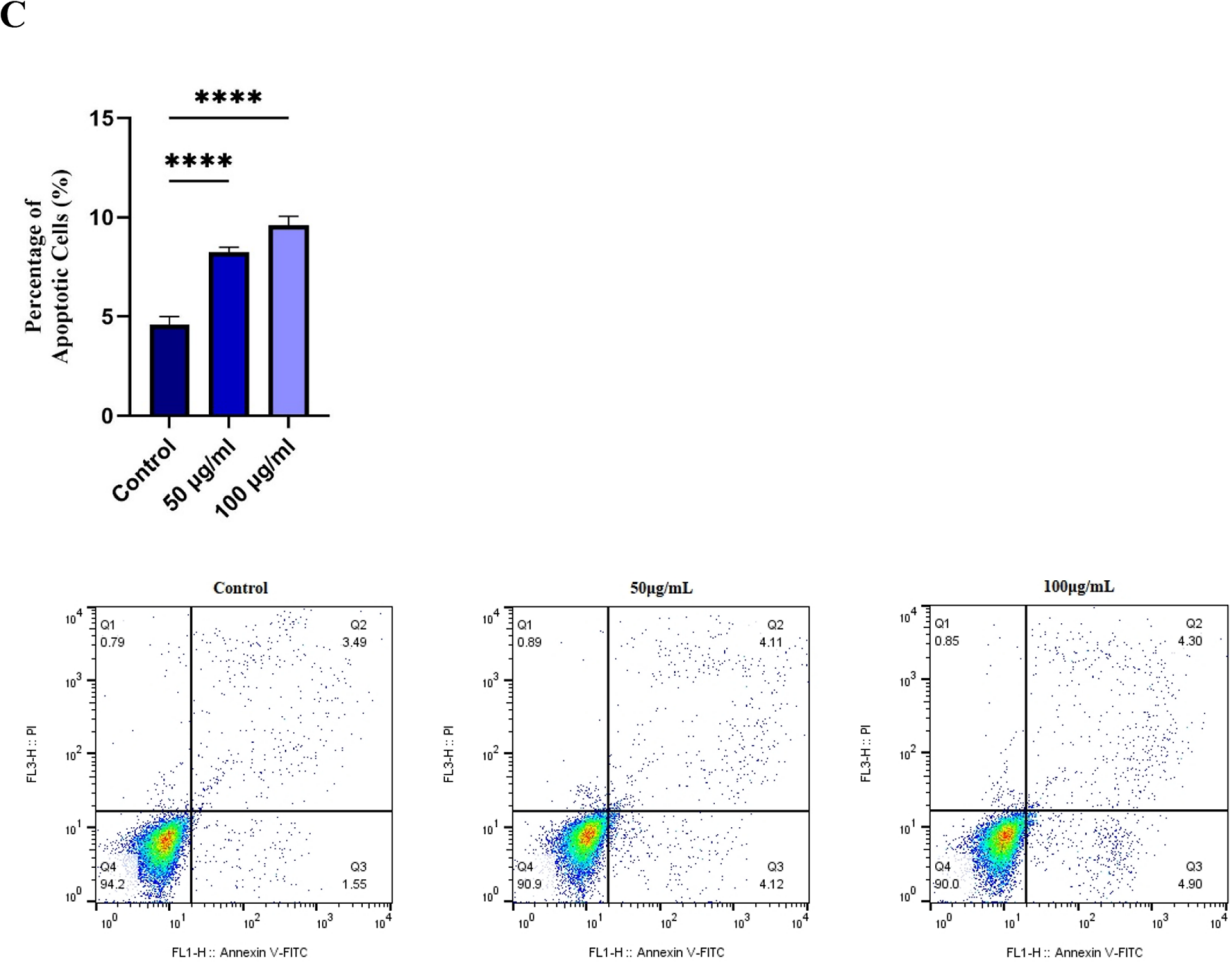
BM-MSC exosomes inhibited cell metabolic activity and cell cycle progression and induced apoptosis in HL-60 cells. (**A**) Cell survival analysis with MTT assay in treated HL-60 cells with 50 and 100 μg/mL of BM-MSC-derived exosomes after 24 and 48 h time intervals. Both concentrations significantly decreased the viability of treated cells compared with the control. (**B**) HL-60 cell cycle progression was blocked in the G0/G1 phase after treatment with 100 μg/mL of BM-MSC exosomes. The histogram demonstrates the cell cycle phases according to the intensity of PI in HL-60 cells after treatment with 50 and 100 μg/mL of BM-MSC exosomes for 24 h. (**C**) The comparison between the impact of different concentrations of BM-MSC exosomes (50 and 100 μg/mL) on HL-60 cells’ apoptosis in 24 h demonstrated a meaningful increase in the apoptosis level of cells treated with both concentrations. Cell apoptosis was examined by flow cytometry and quantitatively analyzed (Q1: Necrosis, Q2: Late Apoptosis, Q3: Early Apoptosis, Q4: Live Cells). *p < 0.05, **p < 0.01, ***p < 0.001, and ****p < 0.0001. *BM-MSCs* Bone marrow-derived mesenchymal stromal cells; *PI* Propidium iodide

HL-60 cells treated with increasing concentrations of BM-MSC exosomes were subjected to MTT assay at two different time intervals. The results demonstrated that treatment with 50 and 100 µg/mL of MSC exosomes significantly decreased the viability of treated cells compared with the control, suggesting the inhibitory effect of MSC exosomes on the metabolic activity of HL-60 cells (Fig. 2A). However, as the effects applied within 24 h were more intense, we chose this incubation time for other tests.

### Effect of BM-MSC exosomes on the cell cycle status of HL-60 cells

To investigate whether the inhibitory effect of MSC exosomes on leukemic cells’ proliferation involved the regulation of the cell cycle, the cell cycle phases of HL-60 cells were examined. In order to do so, leukemic cells were first treated with 0, 50, and 100 µg/mL of MSC exosomes for 24 h. The results presented in Fig. 2B revealed that treatment with 100 µg/mL MSC exosomes significantly elevated the G1 phase population, while they slightly decreased the G2 phase population. In cells treated with 50 µg/mL concentration, no meaningful changes were observed.

### Effect of BM-MSC Exosomes on the Apoptosis of HL-60 Cells

To determine the effect of BM-MSC exosomes on the apoptosis induction in HL-60 leukemic cells, Annexin V-FITC/PI staining was performed. Consistent with the MTT results, BM-MSC exosomes could effectively cause apoptotic cell death in HL-60 cells treated with 50 and 100 μg/mL concentrations of these exosomes (Fig. 2C).

### Effect of BM-MSC exosomes on ROS levels in HL-60 cells

**Fig. 3:**
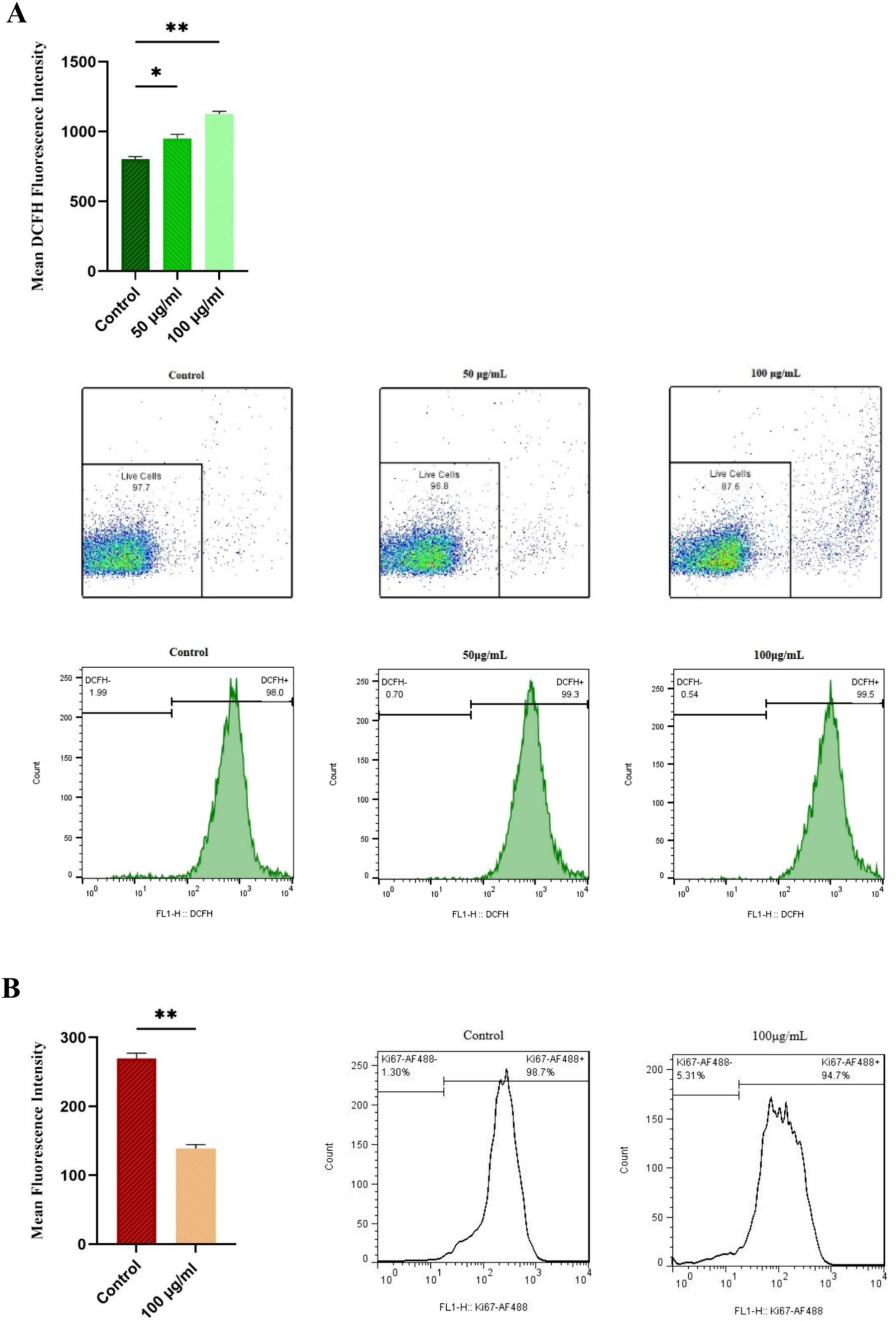
BM-MSC exosomes affected the levels of ROS and Ki67 in HL-60 cells (**A**) Flow cytometry analysis of ROS levels in HL-60 cells after 24 h of treatment with BM-MSC exosomes. The results showed a decrease in the count of live cells and an increase in ROS levels of cells treated with 50 and 100 μg/mL. (**B**) The effect of 100 μg/mL of BM-MSC exosomes on Ki-67 reduction in treated HL-60 cells after 24 h, as detected by flow cytometry

Given the pivotal role of ROS in determining the fate of cells, we evaluated the impact of BM-MSC exosomes on the levels of ROS in HL-60 cells. Based on our findings, compared to the control group, the 50 and 100 μg/mL doses of exosomes strongly upregulate the ROS levels of HL-60 cells (Fig. 3A). Based on the significant inhibition of metabolic activity, arrest in the cell cycle, promotion of apoptosis, and upregulation of ROS levels in HL-60 cells at 100 mg/mL concentration, it was chosen as the working concentration for subsequent experiments.

### Effect of BM-MSC exosomes on the Ki-67 level in HL-60 cells

To examine whether BM-MSC exosomes affect the proliferative capacity of HL-60 cells, we assessed the expression of Ki-67 using flow cytometry analysis. Our results demonstrated that the level of Ki-67 expression in HL-60 cells treated with 100 μg/mL of BM-MSC exosomes was significantly lower than that in the control group (Fig. 3B).

### Effect of BM-MSC exosomes on the gene expression of HL-60 cells

**Fig. 4:**
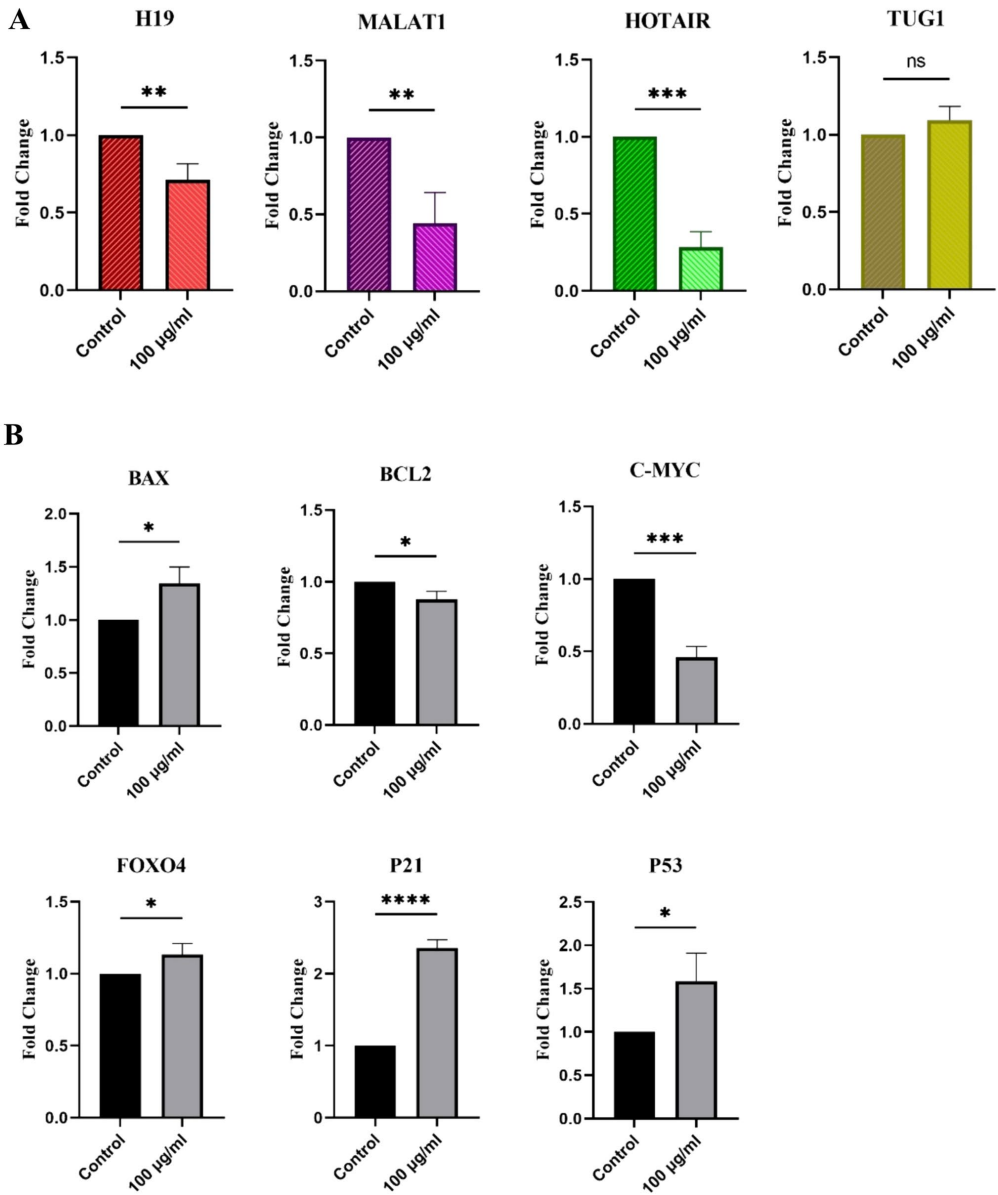
Relative gene expression (**A**) MALAT1, HOTAIR, and H19 lncRNAs were significantly decreased in HL-60 cells due to treatment with 100 μg/mL of exosomes, while TUG1 expression was not affected meaningfully. (**B**) Relative expression of genes involved in cell cycle arrest, apoptosis, and the ROS index in HL-60 cells after treatment with 100 μg/mL of BM-MSC exosomes. While we observed an elevation in p21, p53, BAX, and FOXO4 levels, the expression of BCL2 and c-Myc decreased. The expression levels were normalized with the expression of the GAPDH housekeeping gene. *p < 0.05, **p < 0.01, ***p < 0.001, and ****p < 0.0001 versus control (*n* = 3)

We first determined the expression profiles of MALAT1, HOTAIR, H19, and TUG1 in HL-60 cells after treatment with 100 mg/mL of BM-MSC exosomes for 24 h by qRT-PCR. The resulting data showed that MALAT1, HOTAIR, and H19 expression levels were significantly decreased, and the TUG1 expression level did not change meaningfully in treated cells compared with control cells (Fig. 4A).

In addition, the expression of genes involved in cell cycle arrest, ROS levels, and apoptosis was examined in HL-60 cells. The results of RT-qPCR analysis demonstrated that upon exposure to 100 mg/mL of BM-MSC exosomes, there was a reduction in the expression level of the anti-apoptotic gene, BCL2, coupled with an elevation in the expression of the pro-apoptotic gene, BAX.

The expressions of the cell cycle regulator genes p53 and p21 were also increased after this treatment, while the level of c-Myc was significantly decreased compared with that in the control group.

Finally, we found that the expression of FOXO4, which is involved in the cellular response to increased ROS levels, was elevated (Fig. 4B).

## Discussion

Today MSCs are widely used in cell therapy. Recent studies have revealed that MSCs mostly exert their therapeutic effects in a paracrine way, such as releasing exosomes, rather than in a cell–cell contact manner. MSC-derived exosomes have the ability to mediate MSC interactions with multiple cell types, and the therapeutic application of these extracellular vesicles has demonstrated similar results to MSC transplantation without having the risks of cell therapy [9, 29]. However, according to research articles, depending on the type of cancer, the MSC source, and the way the vesicles are isolated, their effects on cancer cells may differ [28, 30]. In recent years, the administration of MSC-derived exosomes had significant effects on a variety of hematological malignancies, including AML [29].

To reveal the potential of exosomes in cancer therapy, the combination of chemotherapeutic agents with MSC exosomes was investigated. MSC exosomes could increase the sensitivity of leukemic cells to imatinib (IM) by inducing apoptosis, suggesting its advantage in supportive treatment, specifically in the case of chemoresistance [9].

In this study, we isolated and characterized exosomes from human BM-MSCs. Our results revealed that HL-60 AML cells exposed to these exosomes demonstrated partially induced apoptosis and a lower cell viability rate than control cells. We also observed a significant inhibition of cell proliferation pointed out by a reduction in Ki67 expression level and an increase in cell population in the G0-G1 phase, suggesting an arrest in cell cycle progression.

These results were consistent with previous in vitro studies indicating the anti-proliferative and pro-apoptotic impact of BM-MSC derived exosomes on KG-1a and THP-1 leukemic cells, despite the fact that exosome isolation techniques were different [31, 32].

Interestingly, our results were also in accordance with an in vivo study by Jiang et al., which demonstrated that 100 μg of BM-MSC exosomes can significantly increase apoptosis and reduce the cancer load of splenic leukemia. They also observed an inhibition of splenomegaly, which is a typical pathological feature of animal leukemia models, and fewer Ki-67-positive cells in the exosome-treated group than in the control group [1].

Furthermore, we investigated the effect of MSC exosomes on the cell cycle and apoptosis-related genes’ expression. P53 has been proven to be one of the central regulators of apoptotic pathways by regulating various pro- and anti-apoptotic genes in the BCL2 family, such as BAX and BCL2. Additionally, p53 negatively affects the cell cycle by inducing p21, which results in G1/S cell cycle arrest [28, 33]. However, it is worth mentioning that in addition to the transactivation of cell cycle arrest genes, p53-mediated cell cycle arrest is also dependent on the trans-repression of c-Myc oncoprotein, which is a multifunctional transcription factor that stimulates cell growth and proliferation and regulates the cell cycle by repressing p21 [34, 35]. Based on the results of our study, MSC exosomes could increase the expression of p53 and its downstream target gene, p21, and effectively decrease the c-Myc expression level, indicating the induction of cell cycle arrest. These exosomes could also elevate BAX and decrease BCL2 expression, which revealed the induction of the intrinsic apoptosis pathway in treated HL-60 cells. All these findings suggest that the BM-MSC exosomes’ inhibitory effect on AML cells’ progression might be partially mediated by the p53 pathway.

As a by-product of various cellular pathways ROS have the potential to cause DNA damage and induce apoptosis in cells [36]. We demonstrated that the ROS index was elevated in HL-60 cells treated with increasing concentrations of BM-MSC exosomes, which revealed the possible effect of normal exosomes on boosting ROS to damage the genetic content of the cells and lead them to death.

During periods of raised oxidative stress, FOXO4 is activated [37]. Forkhead box O4 (FOXO4) is a member of the FOXO family that reduces cell proliferation by promoting the expression of genes involved in arrest at various phases of the cell cycle and stimulates apoptosis by increasing the expression of genes, such as BAX [38]. We checked the expression level of FOXO4 and found that it was significantly increased, probably in response to elevated levels of ROS. FOXO4-mediated cell cycle arrest, proliferation reduction, and apoptosis induction may be another AML-suppressing mechanism exerted by BM-MSC exosomes.

Recent evidence has suggested that non-coding RNAs are crucial for numerous biological and therapeutic functions mediated by BM-MSC exosomes [3, 31, 32, 39]. Aberrant expression of lncRNAs occurs in all types of cancers including in AML progression and chemoresistance [20, 21].

Metastasis-associated lung adenocarcinoma transcript 1 (MALAT1) is one of the most studied lncRNAs in cancer that has been established to play an oncogenic role and be overly expressed in numerous human cancers, including leukemia [17]. MALAT1 can stimulate tumorigenesis through the PI3K/AKT pathway, Wnt/β-catenin pathway, ERK/MAPK pathway, epithelial–mesenchymal transition (EMT), and angiogenesis [40]. Based on a recent article by Gao et al., MALAT1 upregulation in AML influences apoptosis, proliferation, and drug sensitivity and is associated with poor prognosis, higher WBC and platelet counts, and shorter OS [20]. Our results indicated that BM-MSC exosomes can significantly reduce the level of MALAT1 expression in treated HL-60 cells.

Studies revealed the regulatory role of microRNAs (miRNAs) in the expression of not only 60% of protein-coding genes (by cytoplasmic mRNAs targeting and leading to inhibition of translation or RNA degradation) but also other ncRNAs. miR-9, miR-101, and miR-217, which all can negatively regulate MALAT1 expression, have been found in EVs derived from MSCs [41–45]. Transferring these miRs by our isolated BM-MSC exosomes might be the underlying mechanism of MALAT1 suppression in HL-60 leukemic cells, which requires further investigation.

H19, which is involved in embryonal development and growth control, is another lncRNA involved in solid tumors and hematologic malignancies and is a prognostic and predictive biomarker in AML. H19 exhibits anti-apoptotic and pro-proliferative effects in AML, and its overexpression is correlated with poor chemotherapy response and shorter OS in AML patients [24]. We found that BM-MSC exosomes effectively decreased the expression of H19 at the concentration of 100 μg/mL, which may be mediated through the p53 pathway. Since p53 is a significant tumor suppressor in tumors, it is not surprising that p53 and H19 are mutually counter-regulated. According to articles, not only does p53 suppress the H19 gene promoter activity, but it can also epigenetically repress the expression of H19 in vivo by inducing DNA demethylation of the imprinting control region (ICR) upstream to the H19 gene [46].

LncRNA HOTAIR (HOX Transcript Antisense RNA) has a role in regulating different biological processes in cancers. In hematological malignancies, HOTAIR sustains cell growth and promotes the self-renewal of leukemia stem cells (LSCs) by epigenetic silencing of p15, and its upregulation is associated with higher peripheral leukocyte and BM blast counts, lower hemoglobin counts, and poor DFS [20, 47, 48]. Our findings show that exosomes derived from BM-MSCs are down-regulating HOTAIR expression in leukemic cells. Due to the proven effects of tumor suppressor miR-141 and miR-101-3p on down-regulating HOTAIR [49, 50], and since recent articles demonstrated the exosomal transfer of these miRs from MSCs to various cells [51, 52], hypothetically, they could be responsible for the demonstrated down-regulation of HOTAIR in the HL-60 cells treated with BM-MSC exosomes in our study.

The lncRNA taurine-upregulated gene 1 (TUG1) is a highly conserved functional RNA molecule, and multiple studies have mentioned its role in weakening the sensitivity of AML cells to multiple drugs like cytarabine and adriamycin [20, 53]. Based on our results, BM-MSC exosomes did not have a significant effect on TUG1 expression.

MALAT1, HOTAIR, and H19 lncRNAs affect tumor growth and proliferation by interacting with various microRNAs and transcription factors to regulate cell cycle progression and ki67 level in a way that down-regulation of these lncRNAs is associated with decreased ki67 level, increased p21 expression, G1 cell cycle arrest, and therefore, suppressed proliferation [54–56].

It is also worth mentioning that the aforementioned lncRNAs can affect apoptosis directly by changing the expression of related genes or indirectly through ROS. For example, p53 is one of the main downstream mediators of MALAT1 activity, and its knockdown leads to an elevated apoptosis rate by upregulating p53 and BAX pro-apoptotic proteins and downregulating the anti-apoptotic BCL-2 protein [57, 58].

It is now revealed that ROS level alterations in cancer cells may either trigger or fulfill numerous key functions of lncRNAs, such as orchestrating cancer cells toward apoptotic or proliferation pathways, which aligns with the already established characteristics of ROS, such as activating apoptosis when the level reaches a threshold [59].

As MALAT1, HOTAIR, and H19 have proven to have roles in cancer progression by regulating proliferation, apoptosis, cell cycle progression, and ROS levels as the main biological functions, these lncRNAs are considered proper targets in the treatment of various malignancies, including AML. Also, their down-regulation may be one of the underlying mechanisms of exosome-mediated proliferation suppression, cell cycle arrest, and apoptosis induction, highlighting the potential of these exosomes as supportive therapies for AML.

Since exosomes have been widely offered as supporting therapy for many diseases, including cancer, previously developed manufacturing strategies in academic settings are required to be adapted for scaling up.

Exosome-based clinical trials must comply with good manufacturing practices (GMP). The cell cultivation process, exosome purification process, and exosome quality control are critical issues considered in GMP for exosomes [60]. For example, various types of cells like dendritic cells (DCs), BM-MSCs, and adipose tissue-derived stem cells have been used for GMP exosome production, which specific culture parameters such as growth factors, oxygen conditions, cell density, and cell passage should be considered for each of those cell types [61, 62].

Culture systems in academic settings include static systems like flasks while scaling up strategies will exploit dynamic systems such as bioreactors that provide more efficient exosome production compared to a static system by presenting benefits like improved controls of biological parameters such as CO_2_, O_2_, and pH [60].

Efficient exosome isolation is another approach considered in raising the yield of exosomes. In addition to ultracentrifugation-based methods, many alternative techniques for isolating exosomes have been established, and some of them, such as polymer-based precipitation, ultrafiltration, and size exclusion chromatography, can be used for upscaling exosome production [63–65].

Additionally, standardizing storage procedures of purified exosomes is also a critical step for the clinical application of exosomes. Storage at a temperature of -80°C is recommended as the ideal condition that minimizes the impact on the morphology and content of exosomes [64].

Finally, in order to optimize the efficacy and/or delivery of exosomes in clinical trials, engineering strategies are continuously refined to enhance exosome uptake and cargo. One of the developed strategies to enhance cell targeting is the overexpression of specific viral proteins in exosomes due to their attachment to particular receptors on target cells [61].

This current research article has some limitations. First, the use of one AML cell line. Indeed, more studies on different cell lines and even primary cancer cells are necessary to confirm our results. Additionally, we have mostly focused on the impact of BM-MSC-derived exosomes on the proliferation and apoptosis of AML cells. More functional tests should be performed to have a more general idea about their biological functions. 

Second, the experiments were conducted in an in vitro setting, which, although it offers controlled conditions and allows for detailed mechanistic investigations, may not fully represent the complex interactions and physiological responses that occur within a realistic biological context such as the tumor microenvironment. Further research involving animal models or clinical trials would be necessary to validate these findings and assess their relevance for potential therapeutic interventions.

## Conclusion

The present study reports that exosomes released from BM-MSCs efficiently repress metabolic activity, proliferation, and cell cycle progression in HL-60 leukemic cells, enhance the AML cells' ROS index, and increase their apoptosis ratio. Our results also, for the first time, demonstrate the impacts of BM-MSC exosomes on AML by revealing that these exosomes can decrease the expression levels of MALAT1, HOTAIR, and H19 lncRNAs, which are all involved in AML development and poor prognosis. These findings provide a basis for future clinical applications of BM-MSC exosomes in the management of AML.

## Data Availability

The datasets used and/or analyzed during the current study are available from the corresponding author upon reasonable request.

## Abbreviations

AML: Acute Myeloid Leukemia
LncRNA: Long non-coding RNA
BM: Bone Marrow
ROS: Reactive Oxygen Species
TEM: Transmission Electron Microscopy
DLS: Dynamic Light Scattering
MTT: 3-(4,5-Dimethylthiazol-2-yl)-2,5-Diphenyltetrazolium Bromide
RT-qPCR: Reverse Transcription-quantitative Polymerase Chain Reaction
BAX: Bcl-2-Associated X-protein
BCL-2: B-cell lymphoma 2
FOXO4: Forkhead box O4
c-Myc: Cellular Myc
MALAT1: Metastasis-associated lung adenocarcinoma transcript 1
HOTAIR: HOX Transcript Antisense RNA
TUG1: Taurine-Upregulated Gene 1
EDTA: Ethylenediaminetetraacetic acid
PBS: Phosphate-Buffered Saline
DCFH-DA: Dichlorodihydrofluorescein diacetate
